# Sympathetic Overactivation in the Resistant Hypertensive Phenotype: A Meta-Analysis of Published Studies

**DOI:** 10.1161/HYPERTENSIONAHA.125.24749

**Published:** 2025-06-18

**Authors:** Guido Grassi, Fosca Quarti-Trevano, Cesare Cuspidi, Elias Sanidas, Giuseppe Mancia, Costas Thomopoulos

**Affiliations:** 1 Department of Medicine and Surgery, University Milano-Bicocca, Italy (G.G., F.Q.-T., C.C., G.M.).; 2 IRCCS San Gerardo Hospital, Monza, Italy (F.Q.-T.).; 3 Department of Cardiology, General Hospital of Athens Laiko, Greece (C.C., E.S.).

**Keywords:** baroreflex, blood pressure, heart rate, hypertension, sympathetic nervous system

## Abstract

**BACKGROUND:**

Indirect and direct approaches to assess sympathetic cardiovascular drive have shown that patients with essential hypertension responsive to the blood pressure–lowering effects of antihypertensive drugs are characterized by a pronounced adrenergic overactivity. Whether an emerging clinical hypertensive phenotype such as drug-resistant hypertension (RHT) is also characterized by sympathetic activation and whether its magnitude and underlying pathophysiological mechanisms differ from those of non-RHT is undefined.

**METHODS:**

Among the 54 studies identified providing information in RHT on muscle sympathetic nerve traffic (MSNA), 12 were eligible (508 patients) and meta-analyzed, grouping them based on clinically relevant questions: (1) Is MSNA increased in RHT? (2) Does the magnitude of the sympathetic activation differ from that observed in non-RHT? (3) Are heart rate and plasma norepinephrine valuable surrogate markers of MSNA in RHT? and (4) Is baroreflex-MSNA control impaired?

**RESULTS:**

MSNA was significantly greater in patients with RHT than in normotensive patients (73.2±6.6 versus 46.1±11.1 bursts/100 heartbeats, means±SD; *P*<0.0001) and this was the case also when data were compared with patients with non-RHT (59.8±8.4 bursts/100 heartbeats; *P*<0.001), despite the greater number of antihypertensive drugs. At variance from non-RHT, in RHT, elevated MSNA was unrelated to heart rate and plasma venous norepinephrine. Similar to non-RHT, MSNA in RHT was inversely related to the baroreflex function.

**CONCLUSIONS:**

RHT is characterized by a sustained sympathetic overdrive, significantly greater in magnitude than the 1 detected in non-RHT. Neither heart rate nor norepinephrine are capable of reflecting the marked adrenergic overdrive seen in this condition via MSNA recordings.

**Novelty and Relevance**

**What Is New?**

This meta-analysis is aimed at addressing 3 issues never examined before, that is, (1) the behavior of sympathetic nerve traffic, assessed via clinical microneurography, in resistant hypertension (RHT), (2) the comparison with non-RHT, and (3) the relationships between sympathetic nerve traffic, heart rate, plasma venous norepinephrine, and baroreflex function.

Twelve microneurographic studies for a total of 512 patients were meta-analyzed.

Results show that RHT exhibits a significant increase in sympathetic nerve traffic as compared with the normotensive control state. They also show that the sympathetic activation is significantly greater in magnitude than the 1 detected in non-RHT.

In contrast to what was reported in essential hypertension responsive to the blood pressure–lowering effects of antihypertensive drugs, in RHT no significant relationship was found between sympathetic nerve traffic and surrogate sympathetic markers, such as plasma venous norepinephrine and heart rate. An inverse significant relationship was found with the baroreflex function.

**What Is Relevant?**

Results of the present meta-analysis provide the first evidence that the sympathetic overactivity characterizing essential hypertension is remarkably potentiated in RHT.

Alterations in baroreflex cardiovascular modulation may contribute to this profound sympathetic activation, which can be only in part detectable by norepinephrine and heart rate assessment.

**Clinical/Pathophysiological Implications?**

The marked sympathetic activation detected in RHT may participate in the elevated cardiovascular risk profile of this clinical condition. Its specific detection in RHT also suggests the need to counteract the adrenergic overactivation with pharmacological and nonpharmacological (ie, bilateral renal nerve ablation) interventions capable of exerting sympathoinhibitory effects.

Microneurographic studies based on the direct recording of efferent postganglionic sympathetic nerve traffic (MSNA) to the skeletal muscle vascular district in patients with essential hypertension have provided conclusive evidence that this clinical condition is characterized by a consistent sympathetic activation, thereby strengthening the findings (frequently not univocal) collected by employing indirect approaches to assess sympathetic cardiovascular function, such as the assay of plasma venous norepinephrine and the measurement of resting heart rate (HR).^1–4^ Evidence has been also provided that the sympathetic overdrive (1) is potentiated in patients with essential hypertension in which the high blood pressure (BP) state is associated with other clinical conditions also characterized by an adrenergic overdrive, (2) is detected independently on patients’ age and gender, (3) relates to the elevated BP values and the presence of target organ damage, and (4) characterizes different clinical hypertensive phenotypes, such as white-coat, masked, masked uncontrolled, and refractory hypertension.^3,5–8^

Whether and to what extent another emerging clinical hypertensive phenotype, known as drug-resistant hypertension (RHT), is also characterized by sympathetic overactivation remains unclear. The issue has pathophysiological and clinical relevance, considering the potential therapeutic implications related to the detection of a hyperadrenergic state.^9^ Specifically, it remains unsettled whether RHT displays a sympathetic overdrive of greater magnitude than the 1 detected in the hypertensive states responsive to the BP-lowering effects of antihypertensive drugs. These uncertainties may rely on a variety of factors, including the small sample size of the studies available, frequently dependent on the difficulties intrinsic to the microneurographic technique to obtain stable MSNA recordings with an optimal signal-to-noise ratio in the recruited patients.

The present meta-analysis was conceived to overcome some of the above limitations, by determining MSNA from a large number of studies and thus from a wider population sample. The analysis was extended to the relationships of MSNA with other surrogate neuroadrenergic markers, such as resting HR and plasma venous norepinephrine.^3,10^ In a few studies, analysis was also extended to the relationships of MSNA with arterial baroreceptor dysfunction, which characterizes high BP states including RHT.^10,11^

## Methods

### Data Availability

The data supporting the findings of this study are available from the corresponding author on reasonable request.

### Protocol and Registration

We assessed the difference in MSNA measures between patients with RHT and patients with hypertension without the RHT phenotype, used as controls. The Preferred Reporting Items for Systematic Reviews and Meta-Analyses guidelines were adhered to.^12^ This systematic review was registered at the International Prospective Register of Systematic Reviews database (URL: https://www.crd.york.ac.uk/PROSPERO/; Unique identifier: CRD42025636708).^13^

### Eligibility Criteria

Case-control or cohort studies were eligible whether they provided data for the comparison of MSNA measures between patients with (1) RHT subsequently undergoing renal denervation or carotid baroreflex stimulation versus RHT or non-RHT phenotype used as controls, (2) RHT versus non-RHT, and (3) RHT versus normotension. Differences in systolic/diastolic BP and the number of antihypertensive drugs used were additional mandatory criteria to include studies. In cohort studies of patients with RHT without a control group, a historical cohort of controls was used.^14^ We excluded studies (1) without or incomplete data on MSNA measures, (2) without BP or HR values, and (3) without information about the antihypertensive drug regimen.

### Information Sources and Search

In the first week of September 2024, 3 independent reviewers (G.G., F.Q.-T., and C.T.) performed a systematic literature search in PubMed and the Cochrane Collaboration Library databases to select eligible studies published until August 31, 2024. A search strategy was organized around the setting of RHT and the measure of interest, that is, MSNA. The comparison between the RHT and the non-RHT phenotype was primarily pursued, but additional comparisons between RHT and controls were not excluded. Searching procedures with the appropriate keywords are presented in Table S1.

### Study Selection and Data Collection Process

Titles and abstracts of studies retrieved through database searching were screened by 3 independent reviewers (G.G., F.Q.-T., and C.T.) to identify studies potentially meeting the inclusion criteria. The full text of these potentially eligible studies was retrieved and independently assessed for eligibility by the same 3 reviewers. Any disagreement was resolved through discussion. A standardized, prepiloted form was used to extract data from the included studies. Two reviewer authors (E.S. and C.C.) extracted data independently, while discrepancies were identified and resolved through discussion. For each study, the following information was extracted: publication details (first author’s name, journal, and year of publication), study characteristics (country of conduction and type of study), number of participants, mean age of participants, male sex prevalence, number of antihypertensive drugs, attrition rate, mean systolic/diastolic BP, and mean HR values.

### Risk of Bias in Individual Studies and Quality of Evidence

Two independent reviewers (E.S. and R.F.) assessed the risk of bias in individual studies using the Risk of Bias in Non-randomized Studies - of Exposures (ROBINS-E) tool to reflect the studies’ quality.^15^ The 7 domains of the ROBINS-E tool through which bias may be introduced into outcomes refer to (1) bias because of confounding, (2) bias arising from the measurement of the exposure, (3) bias related to the selection of study participants, (4) bias related to postexposure interventions, (5) bias related to missing data, (6) bias related to the measurement of the outcome, and (7) bias in the selection of the reported result. For each domain, the possible risks of bias judgments are: (1) low risk of bias, (2) some concerns, and (3) high risk of bias. The overall risk of bias judgment for each study, about the examined outcome was defined as low risk of bias if the study was judged to be at low risk of bias for all domains for this result, some concerns if the study was considered to raise some concerns in at least 1 domain for this result, but not to be at high risk of bias for any domain and high risk of bias if the study was judged to be at high risk of bias in at least 1 domain for this result or the study is considered to have some concerns for multiple domains in a way that substantially lowers confidence in the result.

The same 2 reviewers (E.S. and R.F.) rated the quality of evidence separately for each outcome using the grading of recommendations, assessment, development, and evaluation approach.^16^ Rating of the overall quality of evidence for each outcome is based on the judgments about each of the quality of evidence factors assessed (ie, risk of bias, indirectness, inconsistency, imprecision, and other considerations) and quality of evidence grades are defined as high, moderate, low, or very low. Disagreements between the 2 reviewer authors over the risk of bias in individual studies and the quality assessment of the evidence were resolved through discussion, with the involvement of a third reviewer author (G.M.) whenever necessary.

### Outcomes and Data Analysis

The outcomes explored in this meta-analysis were MSNA values, expressed as bursts incidence over time (bursts/min) or as bursts incidence corrected for HR (bursts/100 heartbeats). In the primary analysis, all studies were considered together, and then sensitivity analyses were performed: (1) according to the type of comparison (RHT versus non-RHT, RHT in patients selected to undergo an interventional treatment of hypertension versus RHT with patients chosen not to proceed with an intervention), (2) limited to studies with a control group (ie, excluding cohort studies), and (3) limited to higher quality studies.

Because no individual participant data were available, analyses were performed using the tabular data from the original study publications. Baseline clinical characteristics of the participants, baseline systolic/diastolic BP difference, baseline HR difference, and the difference in the number of drugs between the 2 arms represent the mean of individual study values weighted by participants’ number (ie, weighted average) using the random-effects model. Outcome variables were pooled as standard differences in means with 95% CI. Information on other markers of sympathetic activity such as HR was obtained in all studies, while plasma venous norepinephrine in 4 studies only. Data on baroreflex modulation of MSNA were reported in 3 studies only. The proportion of inconsistency across studies not explained by chance was assessed by the I^2^ index. A random-effects model was used for all analyses because it is the most appropriate when data from studies of the literature with different clinical characteristics are gathered together. The influence of an individual study on pooled effect size was tested by excluding 1 trial at a time: if the point estimate of the combined effect size with a given study excluded lay outside the 95% CI of the overall BP estimate with all available studies, the study in question was considered to have excessive influence. Univariate and multivariate meta-regression analyses with a limited and selective number of covariates (ie, age, the prevalence of male sex, baseline difference in systolic/diastolic BP, HR, and antihypertensive drugs) were conducted to determine whether the modulating effect of clinical variables remains significant. The publication bias was investigated graphically using funnel plots under a random-effects model, Duval, Tweedie’s trim and fill method, and Egger’s regression test. All statistical analyses were done using the Comprehensive Meta-Analysis, version 3 (Biostat; Englewood, NJ). In each analysis, a *P*<0.05 (2-tailed) was considered statistically significant.

## Results

### Study Selection and Characteristics

**Figure 1.**
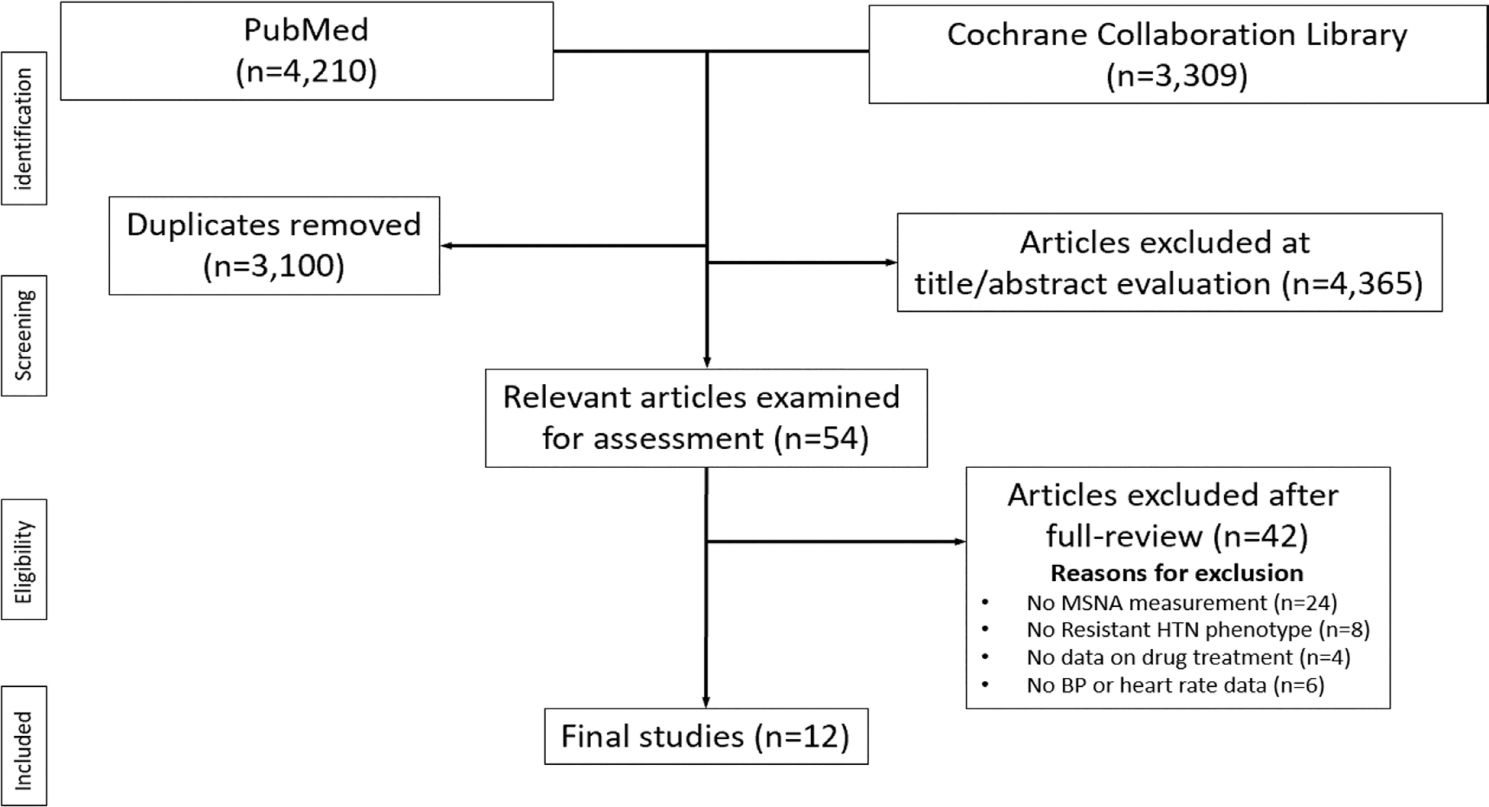
**Flowchart summarizing the methodology for selecting the included studies.** BP indicates blood pressure; HTN, hypertension; and MSNA, muscle sympathetic nerve traffic.

The flow diagram of the study selection process is shown in Figure 1. A total of 7519 records were identified through the database search. After duplicates were removed (n=3100), 4365 records were excluded based on the title and abstract evaluation, while 54 articles were assessed for eligibility at the full-text level. We further excluded 42 studies based on predetermined criteria, and we finally selected 12 studies for a total of 508 patients.^11,17–27^ Recruited patients mean age amounted to 57.4 years, men to 75.2%, systolic/diastolic BP difference to 20.6/11.0 mm Hg, HR difference to 0.5 bpm, difference in the number of antihypertensive drugs to 2. Table 1 summarizes the characteristics of the studies included in the present meta-analysis.^11,17–27^

**Table 1.**
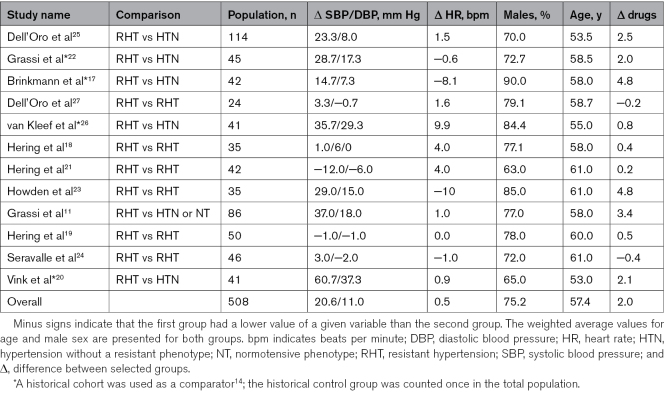
Clinical Characteristics of the Studies Included in the Meta-Analysis

### MSNA Measures in RHT: Primary Analysis

**Figure 2.**
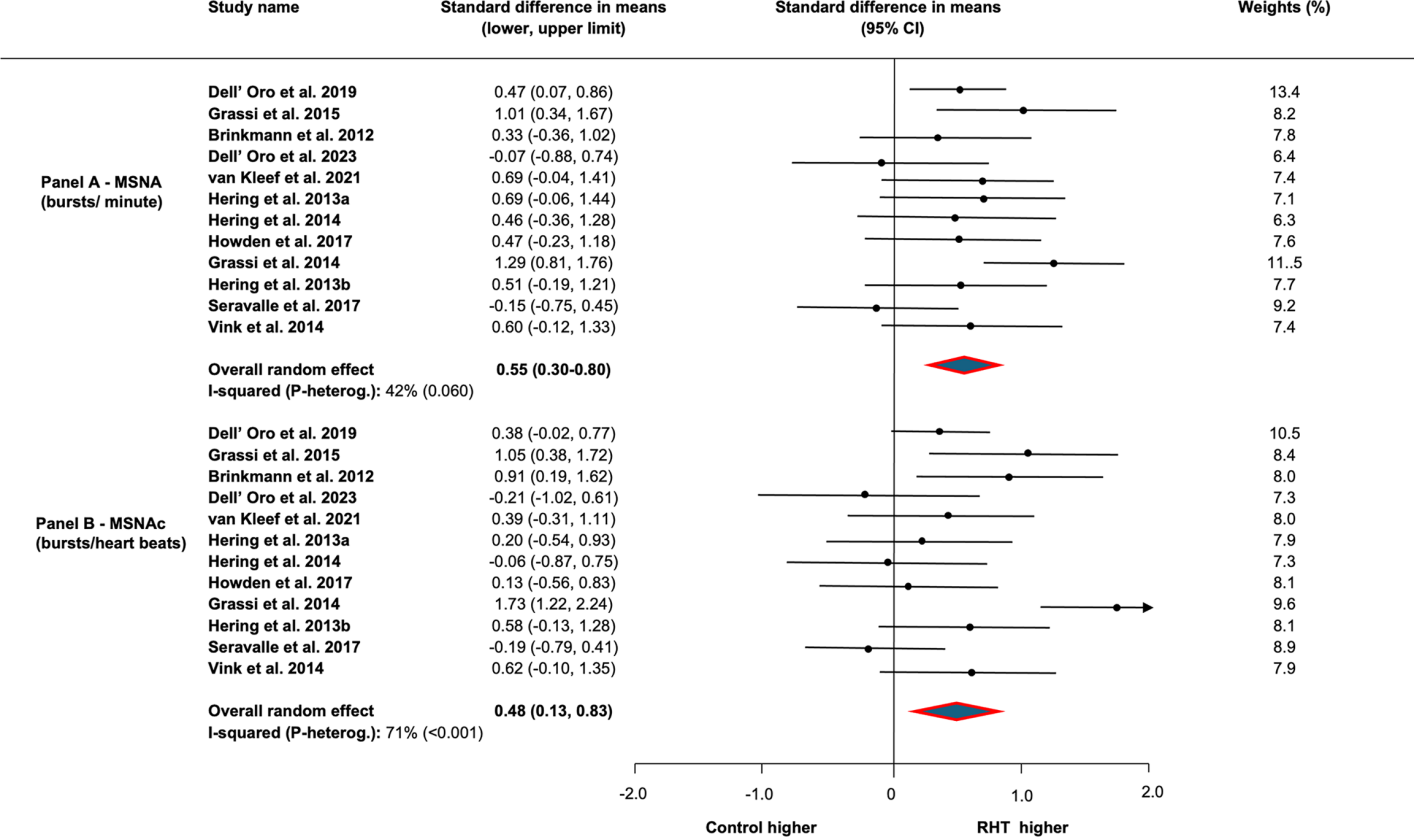
**Mean differences in muscle sympathetic nerve traffic (MSNA) between patients with resistant hypertension (RHT) and patients with essential nonresistant hypertension and normotensive subjects (controls). A**, MSNA expressed as bursts incidence over time (bursts/min). **B**, Muscle sympathetic nerve traffic values corrected for heart rate (MSNAc) expressed as bursts incidence corrected for heart rate (bursts/100 heartbeats).

In all studies, the standard difference in means between groups amounted to 0.55 (95% CI, 0.30–0.50) for bursts/min and 0.48 (95% CI, 0.13–0.83) for bursts/100 heartbeats (Figure 2). The heterogeneity between studies was moderate to significant. No study excessively affected the outcomes according to one-study removed analysis (data not shown). Regarding the unstandardized difference in means, MSNA as bursts/min and as bursts/100 heartbeats amounted to 6.70 (95% CI, 3.16–10.25) and 7.97 (95% CI, 1.84–14.10), respectively.

### Sensitivity Analyses

**Figure 3.**
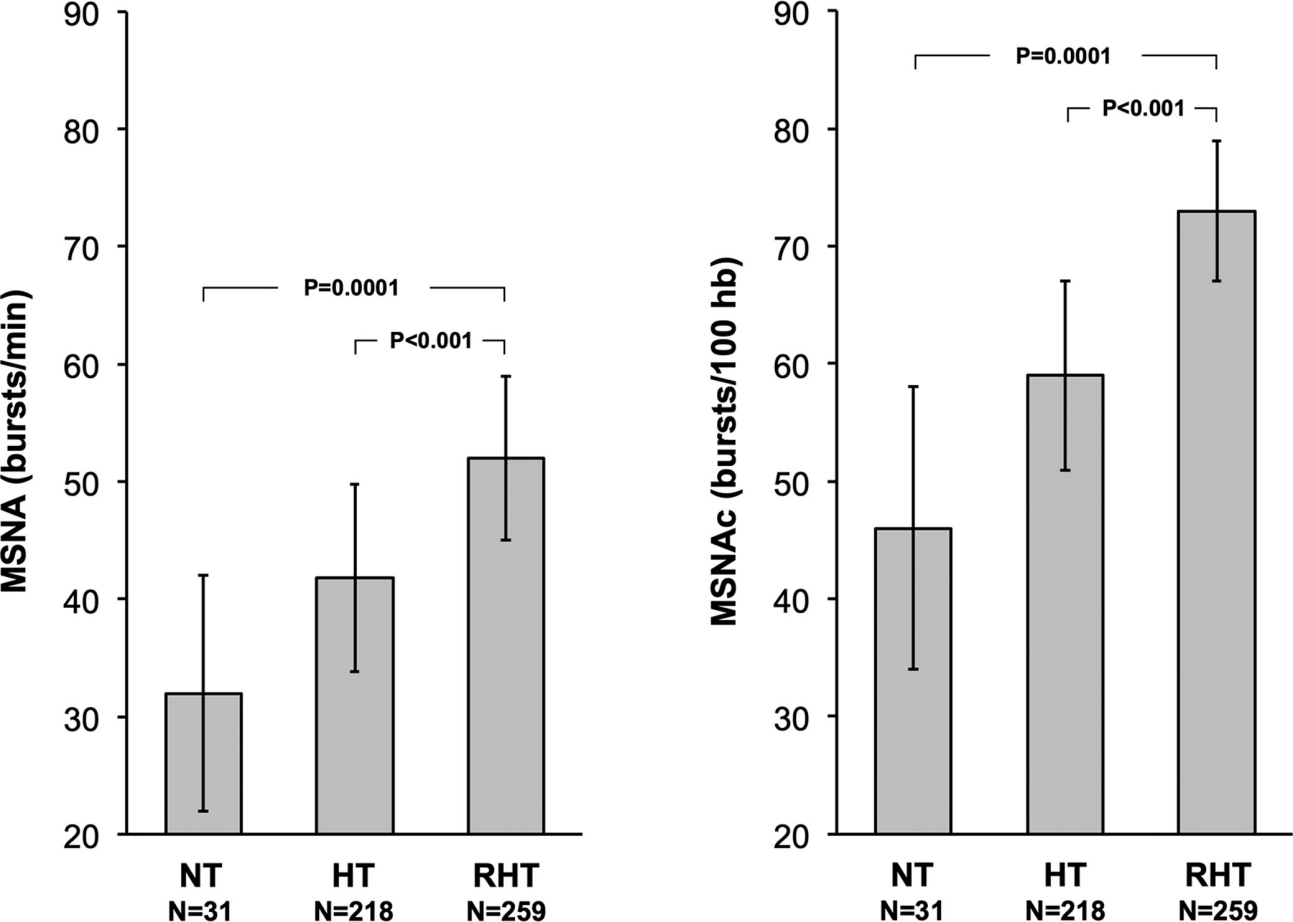
**Muscle sympathetic nerve traffic (MSNA) expressed as bursts frequency over time (bursts/min, left) and as bursts frequency corrected for heart rate (MSNAc; bursts/100 heartbeats, right) in normotensive subjects (NT), nonresistant (HT), and resistant essential (RHT) hypertensives.** Differences between groups were highly statistically significant (always *P*<0.05). Data are shown as means±SD. Data from 508 individuals were evaluated in 12 microneurographic studies.

The comparison between RHT and non-RHT groups demonstrated significantly higher MSNA values, favoring the former group (Figure 3; Table 2). Notably, heterogeneity was downgraded compared with the higher levels of heterogeneity observed in the primary analysis. In Table 2, it is also possible to note that MSNA values between patients with RHT scheduled to undergo interventional treatment (renal denervation) and those not selected for the procedure were not different. By excluding studies in which a historical cohort was chosen as a comparator, only the difference in MSNA expressed as bursts/min remained significant. The analysis, including studies of higher quality, was not different compared with the primary analysis (Table 2).

**Table 2.**
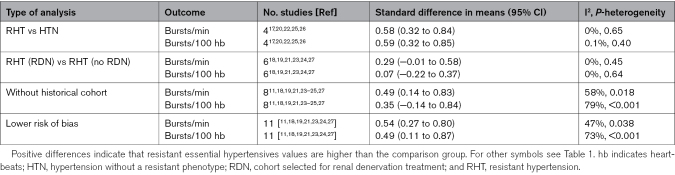
Sensitivity Analysis Limited to Studies With Different Characteristics or Level of Quality

### Meta-Regression Analyses

Univariate meta-regression analyses demonstrated a marginal but significant positive association between MSNA values and the difference in systolic BP between groups. No significant association was detected between resting HR and MSNA values, both when expressed as burst frequency over time (β-standardized coefficient, 0.07; *P*=NS) and as bursts corrected for HR (β-standardized coefficient, 0.44; *P*=NS). This was the case also for the relationships between venous plasma norepinephrine and MSNA values (*r*=0.11; *P*=NS). A significant association between the difference in the number of antihypertensive medications and MSNA in bursts/100 heartbeats was also detected (Table S2). In a multivariable meta-regression model (Table 3), the differences in HR, age, and number of antihypertensive drugs were related to MSNA expressed as bursts/min, but not to MSNA expressed as bursts/100 heartbeats. MSNA values were significantly and inversely related to baroreflex-MSNA function (*r*=−0.34; *P*<0.05) in the 3 published studies evaluating in 35 patients this variable. No data were available to differentiate MSNA activation in controlled versus uncontrolled RHT phenotypes.

**Table 3.**
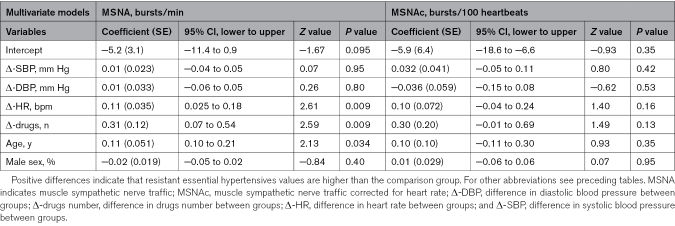
Multivariate Meta-Regression Analyses for the Association of Different Confounders With Outcomes

### Publication Bias, Risk of Bias Within Studies, and Rating of Evidence Quality

As shown in the funnel plots (Figure S1), no publication bias was produced for both outcomes under the random-effects plotting, a finding further suggested by Egger’s regression test. However, the trim and fill test indicated that the imputed effect would be significantly higher than the observed by 25% and 21% for MSNA quantified as bursts/min and as bursts/100 heartbeats, respectively. As indicated in Table S3, the overall risk of bias was low except for concerns about uncontrolled confounding for almost 83% of studies, while 1 study had a low risk of bias and another had a high risk of bias. According to the grading of recommendations, assessment, development, and evaluation approach, the quality of evidence was rated as low or very low for the estimated outcomes. The level of evidence was downgraded because of the observational nature of the study design, the extent of inconsistency, and concerns about publication bias for 1 outcome measure (Table S4).

## Discussion

The present meta-analysis collecting 12 studies, including 259 RHT and 249 normotensive and essential non-RHT controls, is the first ever done evaluating MSNA in RHT and comparing the results with those obtained in nonresistant patients with hypertension. The main study findings can be summarized as follows. First, a direct sensitive marker of the adrenergic cardiovascular drive, such as MSNA, shows a marked and highly significant increase when assessed in RHT as compared with normotensive controls of similar age. Second, the magnitude of the sympathetic activation appears to be much more pronounced, and highly significantly greater, in RHT than in non-RHT patients also of similar age. Third, the differences in adrenergic activation appear only in part related to the difference in BP values between groups. Specifically, in the multivariable analysis, it was found that systolic, but not diastolic, BP values showed a positive significant association with MSNA. Finally, the greater MSNA values found in RHT as compared with non-RHT patients were detectable despite the fact that RHT were treated with a larger number of antihypertensive drugs (on average 2) and thus under a pharmacological treatment with potentially more marked sympathomodulatory effects. It should be worthy of mention, however, that some of the antihypertensive agents used in RHT treatment, such as calcium channel blockers, loop diuretics, and peripheral vasodilatory agents, should have triggered sympathoexcitatory effects.^9^ We cannot thus exclude that, at least in part, the marked sympathetic overactivation detected in RHT may derive from a larger use in these patients of compounds exerting sympathoexcitatory effects.

It can be thus concluded that patients with RHT are characterized by a sympathetic overactivity of much greater magnitude than the 1 characterizing non-RHT and that this is the case regardless of the hypertension severity, the initial or the later stage of the hypertension condition, and the presence of a heightened pharmacological treatment. These conclusions are strengthened by the evidence that data were obtained from a large database ever (about 500 patients).

Several other findings of this study deserve to be discussed. First, as mentioned under Results, no study included in the present meta-analysis provides data allowing to determine whether there is a difference in the MSNA activation between RHT patients with BP uncontrolled or controlled by antihypertensive agents. It should be worthy of mention, however, that in a recent study, we found that in treated patients with essential hypertension, even when displaying satisfactory BP control (BP values <140/90 mm Hg and in some cases <130/80 mm Hg) MSNA remains significantly greater as compared with normotensive controls.^28^ This finding thus indicates that, regardless of BP control, sympathetic activity is not normalized by antihypertensive drug treatment, potentially exerting a harmful impact, particularly in RHT, on the patient’s cardiovascular risk profile.^28^

Second, the results of our study show that in RHT, MSNA was not related to HR values assessed at rest. This finding may imply that at variance from what has been reported in patients with non-RHT essential hypertension responsive to antihypertensive drug treatment and other clinical conditions characterized by sympathetic activation, such as obesity, chronic heart failure, metabolic syndrome, renal insufficiency, and failure,^10,29–33^ in RHT there is a regional heterogeneity of the sympathetic activation, which appears be present at peripheral vascular level but not at the level of the heart. Our findings may also suggest, however, that HR may not represent a sensitive marker of the marked sympathetic overdrive characterizing RHT. This may be because HR is modulated not only by the sympathetic drive but also, and to a greater extent, by the parasympathetic influences on the sinus node.^34^ Based on the data collected in the other clinical conditions mentioned above,^29–33^ this hypothesis can be regarded more likely than the previous one.

Third, in the patients with RHT of the present meta-analysis, venous plasma norepinephrine did not show any significant relationship with MSNA values, in sharp contrast with the significant relationship found between these variables in a previous meta-analysis performed in essential hypertensives responsive to the BP-lowering effects of antihypertensive drugs.^1^ Although the small number of meta-analyzed studies in which plasma norepinephrine was assessed in patients with RHT did not allow to make any firm conclusion, this finding may suggest that venous plasma norepinephrine may have limitations in reflecting the adrenergic cardiovascular overdrive characterizing RHT.

Our meta-analysis shows that in the patients with RHT who underwent evaluation of the baroreflex-MSNA control, this variable was inversely related to resting MSNA values, namely that an impaired modulation of sympathetic drive by arterial baroreceptors (and thus a greater baroreflex dysfunction) is associated with more elevated values of MSNA. This suggests that arterial baroreceptor impairment may be one of the mechanisms involved in the development of the RHT-related marked sympathetic overdrive. However, given the consideration that a similar baroreflex dysfunction has been also reported in the non-RHT phenotype,^1,3,10^ the specificity of this finding to RHT appears unlikely. Finally, this study was not designed to determine the mechanisms through which the sympathetic overactivation found in RHT may participate in determining the sustained BP elevation characterizing this hypertensive phenotype. We can, however, identify 2 pathophysiological processes, mediated by an increase in the sympathetic cardiovascular influences to the arteries and the kidneys, namely the potentiation of the peripheral vasoconstrictor drive and the enhancement of the renal sodium reabsorption process.^3,4,10,35^

## Perspectives

Some limitations and strengths of the current meta-analysis merit to be mentioned. The first limitation, which is shared by all the meta-analytic investigations, includes the fact that the evaluation we did depends on the number, size, and design of the various studies included in the analysis, and this may have potentially affected to some extent the results. The second limitation refers to the fact that comparisons were made without correction for multiple testing, not excluding the possibility that some of the differences may be because of chance. A third limitation refers to the fact that there is some heterogeneity in the included studies about the number of patients, their age, and duration of hypertension, which may have potentially affected the meta-analysis results. Finally, although the presence of a chronic heart failure state concomitant to RHT was an exclusion criterion common to all the studies included in the meta-analysis, based on the clinical and instrumental evidence, the presence of a low-grade heart failure state cannot be entirely ruled out.

The strengths are represented by the fact that the number of studies and of the patients included in the meta-analysis makes the present evaluation one of the largest never done before assessing the role of sympathetic neural factors in RHT development and progression. The clinical implication refers to the evidence that the marked sympathetic activation detected in RHT may participate in the elevated cardiovascular risk profile of this hypertensive phenotype and strengthens the need to counteract the neuroadrenergic activation with pharmacological and device-based therapeutic interventions.^9^ As far as the latter is concerned, it should be worth mentioning that 2 recent meta-analyses performed by our group have conclusively documented the sympathoinhibitory effects exerted by bilateral renal nerves ablation and carotid baroreceptor stimulation in the RHT phenotype.^36,37^

## Article Information

### Sources of Funding

None.

### Disclosures

None.

### Supplemental Material

Tables S1–S4

Figure S1
